# Confidence–performance relationships in prospective and retrospective metacognition across local and global judgments

**DOI:** 10.3389/fpsyg.2026.1900905

**Published:** 2026-10-29

**Authors:** Yuki Otomo, Yousuke Kawachi

**Affiliations:** 1 Department of Psychology, Graduate School of Arts and Letters, Tohoku University, Sendai, Japan; 2 Department of Human Health Sciences, Faculty of Human Health Sciences, Hachinohe Gakuin University, Hachinohe, Japan

**Keywords:** confidence, local-global judgment, metacognition, prospective metacognition, retrospective metacognition

## Abstract

Metacognition is the ability to take an objective perspective on one’s own task performance, both retrospectively and prospectively. Although retrospective metacognition has been extensively studied, prospective metacognition remains poorly understood. Previous studies report that prospective metacognitive accuracy is typically lower than retrospective metacognitive accuracy. This pattern has sometimes been interpreted to suggest that prospective metacognition is inherently limited. The present study examined prospective and retrospective metacognition under different informational conditions, focusing on local judgments concerning individual trials and global judgments concerning multiple trials. Participants performed a perceptual detection task and provided confidence ratings before or after task performance, either for individual trials (local judgments) or for five-trial segments (global judgments). The relationship between task performance and confidence was analyzed using a generalized linear mixed-effects logistic regression model that separated within-participant and participant-condition mean confidence components. Within-participant confidence was positively associated with task performance in both retrospective conditions, with a stronger association for local than global judgments. In contrast, within-participant associations were not statistically significant in either prospective condition and were significantly weaker than their retrospective counterparts. The within-participant slopes did not significantly differ between local and global prospective judgments. Participant-condition mean confidence was positively associated with performance in all four conditions. These findings highlight the need to distinguish associations between participants’ mean confidence and performance from sensitivity to variations in performance within participants. The limited evidence for within-participant prospective sensitivity is consistent with constraints on information available before stimulus presentation, although the present findings do not establish the source of these limitations.

## 1 Introduction

Metacognition refers to the ability to monitor and control one’s own cognitive processes and performance (Flavell, 1979; Nelson and Narens, 1990, 1994). Within this framework, metacognitive monitoring involves assessing one’s own cognitive states and performance, whereas metacognitive control involves regulating cognitive processes on the basis of such assessments (Nelson and Narens, 1990, 1994). The present study focuses on metacognitive monitoring, which can occur at different stages of cognitive processing, including judgments made before, during, and after performance (Nelson and Narens, 1990, 1994). In particular, retrospective metacognition involves reflecting on one’s own experiences and may help guide subsequent behavior, whereas prospective metacognition involves predicting one’s own thinking or performance and may help regulate behavior in advance (Fleming et al., 2016; Siedlecka et al., 2016). Both forms of metacognition play important roles in daily life. For example, prospective metacognitive judgments may help people decide whether and how to engage in an upcoming task, whereas retrospective judgments may help them evaluate their performance and adjust subsequent behavior. More broadly, altered metacognitive processes have also been reported across various psychopathological conditions, including depression and obsessive-compulsive disorder (Sun et al., 2017; Drueke et al., 2023; Irak et al., 2024).

Although there are several recent studies on metacognition in perception (see Rahnev, 2021; Rahnev et al., 2022, for reviews), many of these studies focused primarily on retrospective metacognition. Existing research shows that retrospective confidence is often positively associated with objective performance. In contrast, previous studies on prospective metacognition have reported lower metacognitive accuracy for prospective than retrospective judgments, with prospective accuracy approaching chance levels in some circumstances (Fleming et al., 2016). One explanation for this difference concerns the information available at the time of judgment. Siedlecka et al. (2016) argued that retrospective judgments can incorporate additional information that becomes available during and after the decision, whereas prospective judgments made before the decision cannot incorporate information that becomes available only during or after the decision. Thus, it is important to clarify how the information available at the time of judgment relates to differences between prospective and retrospective metacognition.

In perceptual decision-making research, however, prospective metacognitive judgments have received relatively little attention compared with retrospective confidence judgments. Previous studies have examined prospective metacognition under somewhat different informational conditions. Fleming et al. (2016), for example, asked participants to predict their accuracy before every fifth upcoming trial, allowing them to use information accumulated across previous trials about the stable difficulty of the task. Siedlecka et al. (2016) compared prospective judgments made before and after presentation of the target stimulus, whereas Miyamoto et al. (2021) asked participants to make prospective metacognitive judgments while the stimulus information relevant to subsequent task performance was already available. Thus, prospective judgments in these studies could draw on different amounts or types of task-related information. In the present study, prospective confidence was assessed before presentation of the upcoming stimulus and before the corresponding Type-1 response, without providing trial-specific cues about the upcoming stimulus.

Metacognitive judgments can differ not only in temporal focus, that is, whether they are made before or after task performance, but also in temporal scope, that is, whether they refer to individual trials or to performance across multiple trials. One way to gain a more detailed understanding of prospective metacognition is to examine how it is evaluated across different temporal scopes. Metacognitive judgments are often expressed as confidence ratings. Local judgments refer to the evaluations of individual events, whereas global judgments involve overall evaluations across multiple events. For retrospective judgments, trial-specific information generated during and after a perceptual decision may be more directly relevant to local confidence about that individual trial than to a global evaluation across multiple trials. Previous metacognitive studies have relied predominantly on local trial-by-trial judgments (Cavalan et al., 2023; Katyal and Fleming, 2024; Lee et al., 2021; Rouault et al., 2019; Rouault and Fleming, 2020). However, in everyday life, people are often required to make more global evaluations of how well they are likely to perform, or how well they have performed overall. Examining both local and global judgments may help clarify whether the reduced accuracy of prospective metacognition is a general feature of future-oriented metacognition, or depends on whether performance is evaluated at the level of individual trials or across multiple trials.

Existing evidence is fragmented. Some studies examined prospective metacognition mainly in terms of local judgments (Fleming et al., 2016; Siedlecka et al., 2016), whereas others have addressed both prospective and retrospective metacognition using different levels of judgment within the same study (Zacharopoulos et al., 2022). Other studies have examined how global confidence is formed from multiple local decisions, either in general metacognitive judgments (Rouault et al., 2019) or in perceptual decision-making (Lee et al., 2021). However, these studies have not directly compared prospective and retrospective metacognition at both local and global levels within the same perceptual task. It therefore remains unclear whether prospective disadvantages are expressed similarly across local and global judgments.

Another unresolved issue concerns the aspect of metacognitive judgment that gives rise to prospective disadvantages. If prospective metacognition is weaker than retrospective metacognition, this disadvantage may be expressed in multiple components of metacognitive judgment, particularly metacognitive sensitivity and bias. Distinguishing between these components is important, because poorer metacognitive performance does not, in itself, reveal whether prospective judgments are less related to actual performance, more biased, or both. One possibility is that prospective confidence draws on less diagnostic information than retrospective confidence, because retrospective judgments can incorporate information that becomes available during and after the decision. However, the extent to which prospective confidence predicts subsequent performance should depend on how informative the available cues are about the judgment target. Moreover, differences in mean confidence between participants may correspond to differences in their overall performance even when confidence does not reliably track performance variation within participants.

The nature of the available information may also differ between global and local judgments. Because both types of prospective judgment are made before the upcoming stimulus and the corresponding Type-1 response, they are made without information from either the stimulus or the response. However, the information available for making a prediction may differ between these judgment levels. Local prospective judgments may be influenced by momentary internal states, such as the observer’s current attentional or motivational state, as well as recent task experience, whereas global prospective judgments may rely more strongly on information accumulated across previous trials about the general characteristics or difficulty of the task. If these sources differ in their diagnostic value for the respective judgment targets, the confidence–performance relationship may also differ between global and local prospective judgments.

The present study addressed these issues by comparing prospective and retrospective metacognitive judgments at both local and global levels in a perceptual detection task. Based on previous findings, we expected prospective metacognitive accuracy to be lower than retrospective metacognitive accuracy. We also expected the confidence–performance relationship to be strongest for retrospective local judgments. For prospective judgments, we expected confidence to show a positive relationship with subsequent task performance, but expected this relationship to be weaker than that for retrospective confidence, despite the absence of stimulus- and response-related information. In contrast, we did not have a clear directional prediction for the difference between prospective global and local judgments, because it was unclear whether the information available before task performance would be more diagnostic of performance at the local or global level. To clarify the level at which confidence was associated with performance, our analyses distinguished within-participant variation in confidence across observations from differences in mean confidence between participants within each condition. We also examined metacognitive bias to determine whether participants’ tendency to overestimate or underestimate their actual performance differed across judgment conditions.

## 2 Materials and methods

### 2.1 Participants

A total of 28 participants [10 males and 18 females; mean age = 21.96, standard deviation (SD) = 1.71] participated in the study. Participants were recruited from the university community through mailing lists and classroom announcements. Participants were required to have normal or corrected-to-normal vision and no difficulty engaging in activities in dark and enclosed environments. The required sample size was determined *a priori* using G*Power 3.1.9.7 (*F*-tests: ANOVA: repeated measures, within factors), assuming an alpha level of 0.05, a desired power of 0.80, and an expected effect size of Cohen’s *f* = 0.25. The sample size analysis indicated that 24 participants were required. To allow for possible exclusions, 28 participants were recruited. Before data collection, we established an *a priori* exclusion criterion whereby participants would be excluded if they used only a single confidence-rating value throughout the task. Data from sessions conducted under incorrect program settings were also excluded from analysis. Based on these criteria, two participants were excluded prior to analysis: one because only a single confidence rating was used throughout the task, and the other because the experiment was conducted under incorrect program settings. The final sample comprised 26 participants. This study trials were approved by the Ethical Committee of the Graduate School of Arts and Letters, Tohoku University (IDs: 2022_03_23_Wed_17_00_06, 2023_03_23_Thur_20_36_39), and conducted in accordance with the principles of the Declaration of Helsinki. Written informed consent was obtained from all the participants.

### 2.2 Stimuli

Six Gabor patches were arranged in a circular array at an eccentricity of 6.9° from a central fixation point on a uniform gray background (71.89 cd/m^2^). The fixation point comprised a black square (0.2 degrees per side; 0.41 cd/m^2^) with a smaller white square (0.1° per side; 169.20 cd/m^2^) at its center. Each Gabor subtended at a 1.4° visual angle, had a spatial frequency of 2.2 cycles per degree, and was vertically oriented. The non-target Gabors had a contrast of 20%, whereas the contrasts of the target Gabors were individually titrated for each participant using a 1-up, 2-down staircase procedure. The staircase started at 80% contrast, with contrast adjusted in 3% steps over a range of 23%–80%, and terminated after 10 reversals. If the downward step would have reduced contrast to below 23%, the contrast was fixed at 23%. This procedure estimated the contrast corresponding to approximately 71% accuracy.

### 2.3 Procedure

The experimental stimuli were created using Cogent 2000 and Cogent Graphics running in MATLAB R2021b (MathWorks Inc., Natick, MA, USA). Stimulus generation and experimental control were performed on a Windows PC (LEVEL-G04A-LCiX8K-XAXH, iiyama, Tokyo, Japan), and the stimuli were presented on a monitor (ZOWIE XL2546K; resolution 1920 × 1080 pixels; refresh rate 120 Hz, BenQ Japan, Tokyo, Japan). The participants were seated in a dark room with their heads stabilized by a chin rest positioned 57.3 cm from the monitor.

The experimental paradigm was adapted from Fleming et al. (2010), with modifications to the response format and confidence-rating procedure. Fleming et al. (2010) used a two-alternative forced-choice task with Gabor stimuli, whereas the present study used a Yes/No detection task. Criterion-related effects are not necessarily artifacts to be eliminated and may be meaningful depending on the research question (Peters et al., 2016). We therefore did not specifically design the Type-1 task to minimize such effects. Within this detection format, we examined both components of metacognitive judgment in the present study: the relationship between confidence and task performance (metacognitive sensitivity) and the discrepancy between confidence and actual accuracy (metacognitive bias). These measures were examined across prospective and retrospective judgments and across global and local levels. The multiple-Gabor display and variable target location were retained to preserve the basic spatial structure of the established perceptual metacognition paradigm used by Fleming et al. (2010).

Participants were asked to judge whether a target Gabor patch was present among six Gabor patches arranged in a circular array around a central fixation point, while maintaining fixation at the center of the screen. In Block A (Figure 1A), participants provided a global prospective confidence rating once every five trials before the stimulus presentation, indicating how confident they were in their upcoming performance over the next five trials. Ratings were inputted on a six-point scale (0%, 20%, 40%, 60%, 80%, 100%) using the keyboard. The use of a six-point scale was informed by Fleming et al. (2010), who used a six-point confidence scale in a perceptual decision task. Percentage labels (0%, 20%, 40%, 60%, 80%, and 100%) were used so that both local and global confidence judgments could be expressed in terms of estimated accuracy. After the rating, the stimulus array was presented for 200 ms, and the participants indicated whether the target was present by pressing the left or right arrow key. Following each trial, the participants provided a local retrospective confidence rating using the same six-point scale and keyboard response to indicate how confident they were that their response to that trial had been correct. Both the confidence ratings and detection responses were self-paced. In Block B (Figure 1B), the participants performed the same detection task as in Block A, except that a local prospective confidence rating was obtained before each trial, whereas a global retrospective confidence rating was obtained once every five trials, after the detection response for those five trials. Blocks A and B each comprised 300 trials with a short break every 100 trials. Target-present and target-absent trials were randomized across all trials. In both blocks, each trial was followed by a 500 ms blank interval, and each block was preceded by a 60-trial practice session. No accuracy feedback was provided during either the practice sessions or the main experimental blocks (Blocks A and B). During practice, the experimenter monitored participants’ responses and accuracy to ensure that they adequately understood the task before proceeding to the main experimental block. After completing each block, participants took part in a brief debriefing interview about their task strategies, subjective experiences, and any questions they had about the experiment. The order of Blocks A and B was counterbalanced across participants, and the two blocks were completed on separate days. Each block lasted approximately 60 min, and participants received an Amazon gift card worth JPY 1,000 for each session. All experimental factors were manipulated within participants. Each participant completed a total of 600 trials (300 trials per block), providing repeated observations across all experimental conditions.

**FIGURE 1:**
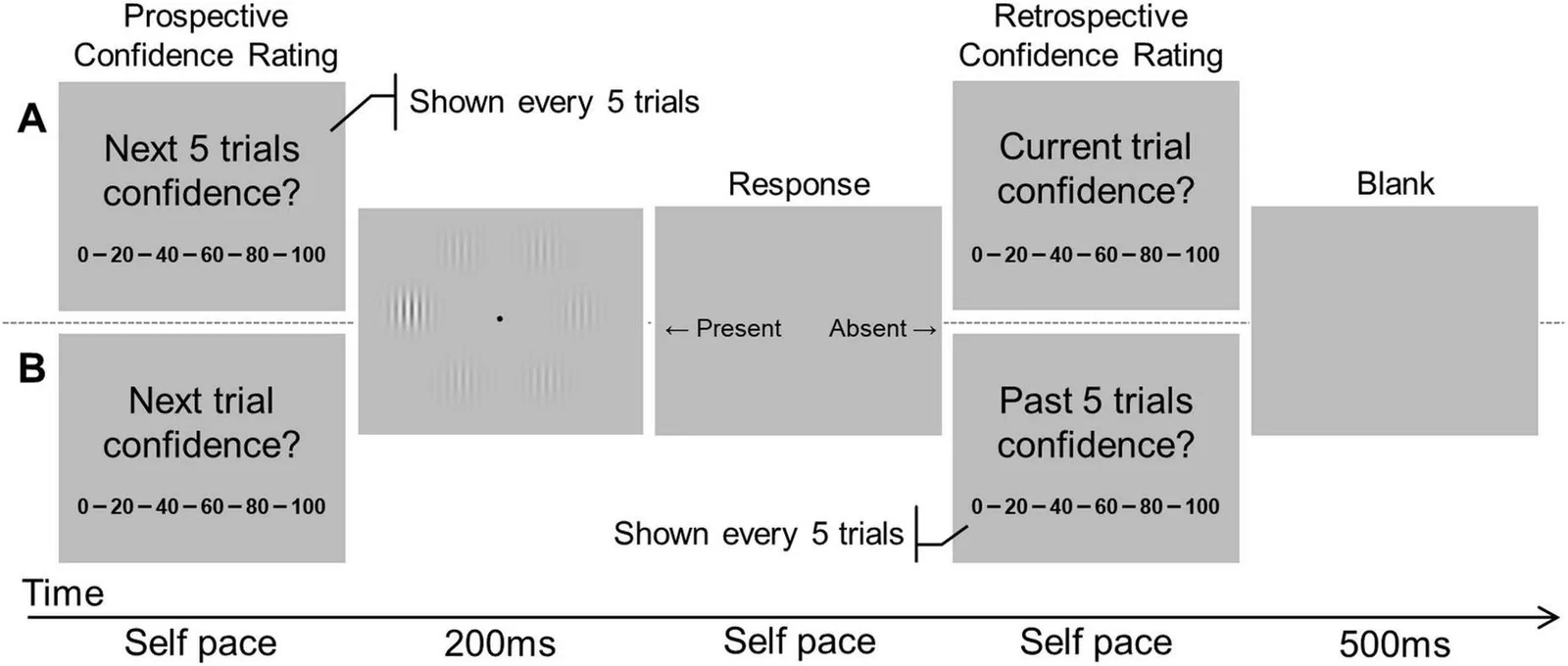
Experimental design. **(A)** Block A consisted of the prospective–global and retrospective–local conditions. **(B)** Block B consisted of the prospective–local and retrospective–global conditions. Global confidence ratings were obtained every five trials in both blocks, whereas local confidence ratings were obtained on every trial. Confidence was rated on a scale from 0 to 100.

### 2.4 Statistical analyses

Each participant completed 300 trials each in Blocks A and B. In the global condition, one confidence judgment was obtained for each five-trial segment, and the detection performance was coded as the number of correct responses out of the corresponding five trials; this method yielded 60 observations per participant per block. In the local condition, one confidence judgment was obtained for each trial, and the detection performance was coded as either correct or incorrect. To reduce any direct influence between local and global judgments, the data for the local condition entered into the analysis were limited to the remaining 240 trials per participant per block, after excluding trials in which global judgments were made.

The main analysis used a generalized linear mixed model (GLMM) with a binomial distribution and logit link function. The number of correct responses was treated as the outcome variable and the number of trials contributing to each observation was specified as the binomial denominator: five for global observations and one for local observations.

To distinguish within-participant variation in confidence across observations from differences in mean confidence between participants within each condition, confidence ratings were decomposed into two components. The within-participant component was defined as each confidence rating minus the participant’s mean confidence in the corresponding temporal focus × temporal scope condition. The participant-condition mean component was defined as the participant’s condition-specific mean confidence minus the overall mean confidence for the corresponding condition. Both components were divided by the same standard deviation, calculated from all original confidence-rating observations across participants and conditions, and were treated as continuous predictors.

Both confidence components, temporal focus (prospective vs. retrospective), temporal scope (global vs. local), and the interactions of each confidence component with temporal focus and temporal scope, including the three-way interactions, were included as fixed effects. The interaction between temporal focus and temporal scope was also included. Block order was included as an additional fixed effect to account for potential order effects. Random intercepts and random slopes for the within-participant confidence component were specified for each participant. The GLMM was specified using the following Wilkinson notation:

number_correct ∼ confidence_within * temporal_focus * temporal_scope + confidence_mean * temporal_focus * temporal_scope + block_order + (1 + confidence_within | subject).

Here, confidence_within and confidence_mean denote the scaled within-participant and participant-condition mean components, respectively. Simple slopes were estimated for each component in each of the four conditions. Holm corrections were applied separately to the four tests of slopes against zero and the six pairwise slope comparisons for each component.

Complementary analyses using the type 2 receiver operating characteristic curve (AUROC2) and Goodman–Kruskal’s gamma, together with the original GLMM using undecomposed confidence ratings, are described in the Supplementary material.

As an exploratory follow-up analysis, we used LMMs to examine whether prospective confidence was associated with recent confidence ratings and task performance, summarized as means over preceding observations. Separate models were fitted for the prospective–global and prospective-local conditions, with current prospective confidence as the dependent variable. Only observations preceding the current prospective judgment were used to construct the history predictors. The specific model formulations are presented with the follow-up analyses in the section “3 Results.”

To further characterize the metacognitive judgments, we computed metacognitive bias indices for each participant and condition. Metacognitive bias was defined as the difference between the mean confidence and mean performance for each participant and condition. Confidence and performance were expressed on the same 0–1 scale before calculating the differences. Values closer to zero indicate better calibration, with positive and negative values reflecting over- and underconfidence, respectively. To examine differences in metacognitive bias across conditions, we fitted a linear mixed-effects model (LMM), with metacognitive bias as the dependent variable. Temporal focus (prospective vs. retrospective), temporal scope (global vs. local), and their interaction were included as fixed effects, and participant was included as a random intercept. The LMM was specified using the following Wilkinson notation:

metacognitive bias ∼ temporal focus * temporal scope + (1 | subject).

The main effect of each factor was tested using a marginal contrast averaged across the levels of the other factor. Statistical analyses were performed using MATLAB R2021b (MathWorks, Natick, MA, USA), with the *fitglme* function for GLMMs and the *fitlme* function for LMMs.

ChatGPT (GPT-6, OpenAI^1^) was used to assist in developing and refining MATLAB analysis scripts. The authors reviewed the AI-assisted code and analytical outputs.

## 3 Results

Before examining metacognitive performance, we assessed task accuracy and target Gabor contrast across blocks and block orders. For mean accuracy, the main effect of block was not significant [*F*(1, 24) = 0.250, *p* = 0.621, partial η^2^ = 0.010], and the main effect of block order was also not significant [*F*(1, 24) = 0.052, *p* = 0.821, partial η^2^ = 0.002]. Although the interaction between block and block order was significant [*F*(1, 24) = 5.267, *p* = 0.031, partial η^2^ = 0.180], none of the corresponding simple effects was significant. For target Gabor contrast, significant main effects of block [*F*(1, 24) = 11.053, *p* = 0.003, partial η^2^ = 0.315] and block order [*F*(1, 24) = 4.982, *p* = 0.035, partial η^2^ = 0.172], as well as a significant interaction [*F*(1, 24) = 7.184, *p* = 0.013, partial η^2^ = 0.230], were observed. However, because target contrast was individually adjusted to maintain approximately comparable task performance, these differences were not accompanied by a systematic difference in task accuracy between blocks. Detailed descriptive statistics are provided in Supplementary Table 1.

### 3.1 The relationship between confidence and task performance

We tested whether prospective and/or retrospective confidence ratings predicted task performance using a generalized linear mixed-effects model (GLMM). Confidence was decomposed into within-participant and participant-condition mean components, which were entered simultaneously into the model. For the within-participant component, a significant interaction with temporal focus was observed (β = 0.214, standard error (*SE*) = 0.058, *t* = 3.690, *p* < 0.001), indicating that the relationship between within-participant variations in confidence and task performance differed between prospective and retrospective judgments in the global condition. The three-way interaction among the within-participant confidence component, temporal focus, and temporal scope was also significant (β = 0.310, *SE* = 0.082, *t* = 3.807, *p* < 0.001), indicating that this difference varied across temporal scopes. In contrast, neither block order (*p* = 0.972) nor the interaction between the within-participant confidence component and temporal scope at the prospective reference level (*p* = 0.564) was significant. To interpret these interactions, we conducted follow-up analyses of simple slopes.

First, we examined the simple slope of the within-participant confidence component for each condition (Holm-corrected *p*-values; Table 1). This component was positively associated with task performance in the retrospective–global condition (β = 0.248, *SE* = 0.039, *z* = 6.346, *p* < 0.001, odds ratio (*OR*) = 1.281, 95% confidence interval (CI) [1.187, 1.383]) and the retrospective–local condition (β = 0.596, *SE* = 0.039, *z* = 15.307, *p* < 0.001, *OR* = 1.816, 95% CI [1.682, 1.960]). However, the associations were not significant in the prospective–global condition (β = 0.034, *SE* = 0.052, *z* = 0.655, *p* = 0.513, *OR* = 1.035, 95% CI [0.934, 1.146]) or the prospective–local condition (β = 0.072, *SE* = 0.051, *z* = 1.417, *p* = 0.313, *OR* = 1.075, 95% CI [0.973, 1.188]) (Figure 2A).

**TABLE 1:** Simple slopes of within-participant and participant-condition mean confidence predicting task performance across metacognitive conditions.

Metacognitive condition		β	*SE*	*z*	OR	95% CI	Holm-corrected *p*
Within-participant confidence component	Prospective–global	0.034	0.052	0.655	1.035	0.934–1.146	0.513
	Prospective–local	0.072	0.051	1.417	1.075	0.973–1.188	0.313
	Retrospective–global	0.248	0.039	6.346	1.281	1.187–1.383	<0.001
	Retrospective–local	0.596	0.039	15.307	1.816	1.682–1.960	<0.001
Participant-condition mean component	Prospective–global	0.191	0.052	3.665	1.211	1.093–1.341	<0.001
	Prospective–local	0.260	0.054	4.794	1.297	1.166–1.443	<0.001
	Retrospective–global	0.323	0.061	5.258	1.381	1.224–1.557	<0.001
	Retrospective–local	0.318	0.070	4.557	1.374	1.198–1.575	<0.001

β, log-odds coefficient; *SE*, standard error; OR, odds ratio; CI, confidence interval. The within-participant component represents deviations from each participant’s mean confidence in each condition. The participant-condition mean component represents participant-specific condition means centered on the corresponding condition mean across participants. Both components were scaled by the same pooled standard deviation of the original confidence ratings and entered simultaneously into the GLMM. Holm correction was applied separately to the four simple-slope tests within each component. Confidence intervals are unadjusted.

**FIGURE 2:**
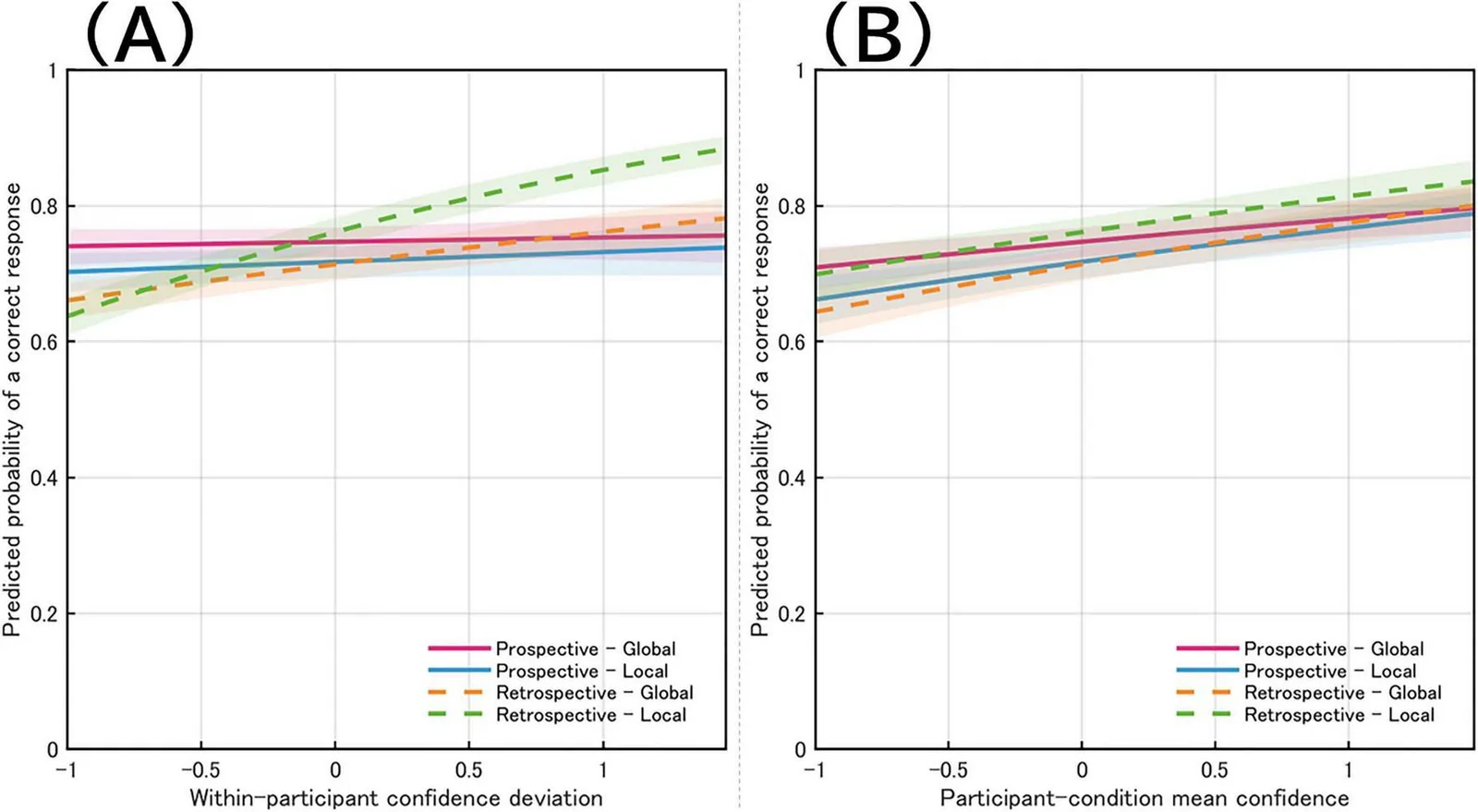
Model-predicted relationships between confidence components and task performance. **(A)** Within-participant confidence component. **(B)** Participant-condition mean confidence component. Both components were scaled by the same overall standard deviation of confidence ratings across participants and conditions. Lines represent fixed-effect predictions from the decomposition GLMM, and shaded bands indicate 95% confidence intervals. For each panel, the other confidence component was held at zero, and block order was fixed at the first-session level. Solid and dashed lines indicate prospective and retrospective judgments, respectively; colors distinguish the four temporal focus × temporal scope conditions. The horizontal-axis ranges differ between panels.

We then conducted Holm-corrected pairwise comparisons of within-participant confidence slopes across conditions. The slope was significantly shallower in prospective–global than in retrospective–global (β difference = −0.214, *SE* = 0.058, *z* = −3.690, *p* = 0.001, OR ratio = 0.808, 95% CI [0.721, 0.905]). Retrospective–global also showed a significantly shallower slope than retrospective–local (β difference = −0.349, *SE* = 0.047, *z* = −7.480, *p* < 0.001, OR ratio = 0.706, 95% CI [0.644, 0.773]). Prospective–local showed a significantly shallower slope than retrospective–local (β difference = −0.524, *SE* = 0.057, *z* = −9.228, *p* < 0.001, OR ratio = 0.592, 95% CI [0.530, 0.662]). The slopes did not significantly differ between prospective–global and prospective–local (β difference = −0.038, *SE* = 0.066, *z* = −0.576, *p* = 0.564).

For the participant-condition mean component, simple slopes were positive and significant in all four conditions (all Holm-corrected *ps* < 0.001; Table 1). Thus, participants with higher mean confidence within a condition tended to show higher task accuracy. None of the pairwise differences between these slopes was significant after Holm correction (all *ps* ≥ 0.204) (Figure 2B). The within-participant relationship between confidence and task performance was the strongest for retrospective judgments in the local condition. The within-participant prospective confidence slopes did not significantly differ between the global and local conditions. Neither prospective within-participant slope differed significantly from zero, although participant-condition mean confidence was positively associated with performance in both prospective conditions.

To further assess the confidence–performance relationship using established measures of metacognitive sensitivity, we additionally calculated AUROC2 and Goodman–Kruskal’s gamma (Fleming and Lau, 2014). Both measures showed stronger confidence–performance relationships for retrospective than for prospective judgments, with the difference between temporal foci being more pronounced for local than for global judgments. Prospective AUROC2 did not significantly differ from 0.50, and prospective gamma did not significantly differ from zero, in either the global or local condition. These findings were consistent with the absence of statistically significant within-participant prospective confidence–performance associations in the decomposition GLMM. Detailed results, together with those of the original pooled GLMM using undecomposed confidence ratings, are reported in the Supplementary material.

### 3.2 Information underlying prospective metacognition

Although the decomposition GLMM did not identify significant within-participant prospective confidence–performance associations, prospective confidence may nevertheless be systematically related to recent experience. We therefore conducted exploratory follow-up analyses to examine whether current prospective confidence was associated with previous confidence and accuracy, and whether the same historical information was associated with subsequent task performance. Separate LMMs for confidence and binomial GLMMs for performance were fitted for the prospective–global and prospective-local conditions, with history predictors decomposed into within-participant and participant-mean components. Detailed model specifications and full results are provided in the Supplementary material.

In both prospective conditions, current confidence was positively associated with within-participant variations in recent local confidence and confidence from the preceding global observation (all Holm-corrected *ps* < 0.001). Recent local accuracy was also positively associated with prospective-local confidence (*p* < 0.001), whereas the remaining within-participant accuracy-history coefficients were not significant after correction. In the subsequent performance GLMMs, none of the within-participant history predictors were significant in either prospective condition (all Holm-corrected *ps* ≥ 0.551). Thus, prospective confidence was consistently associated with confidence history, but these analyses did not identify significant within-participant associations between the examined historical information and subsequent performance.

### 3.3 Prospective and retrospective metacognitive bias

Figure 3 shows the distributions of confidence ratings across the six response options (0%, 20%, 40%, 60%, 80%, and 100%) for each judgment condition.

**FIGURE 3:**
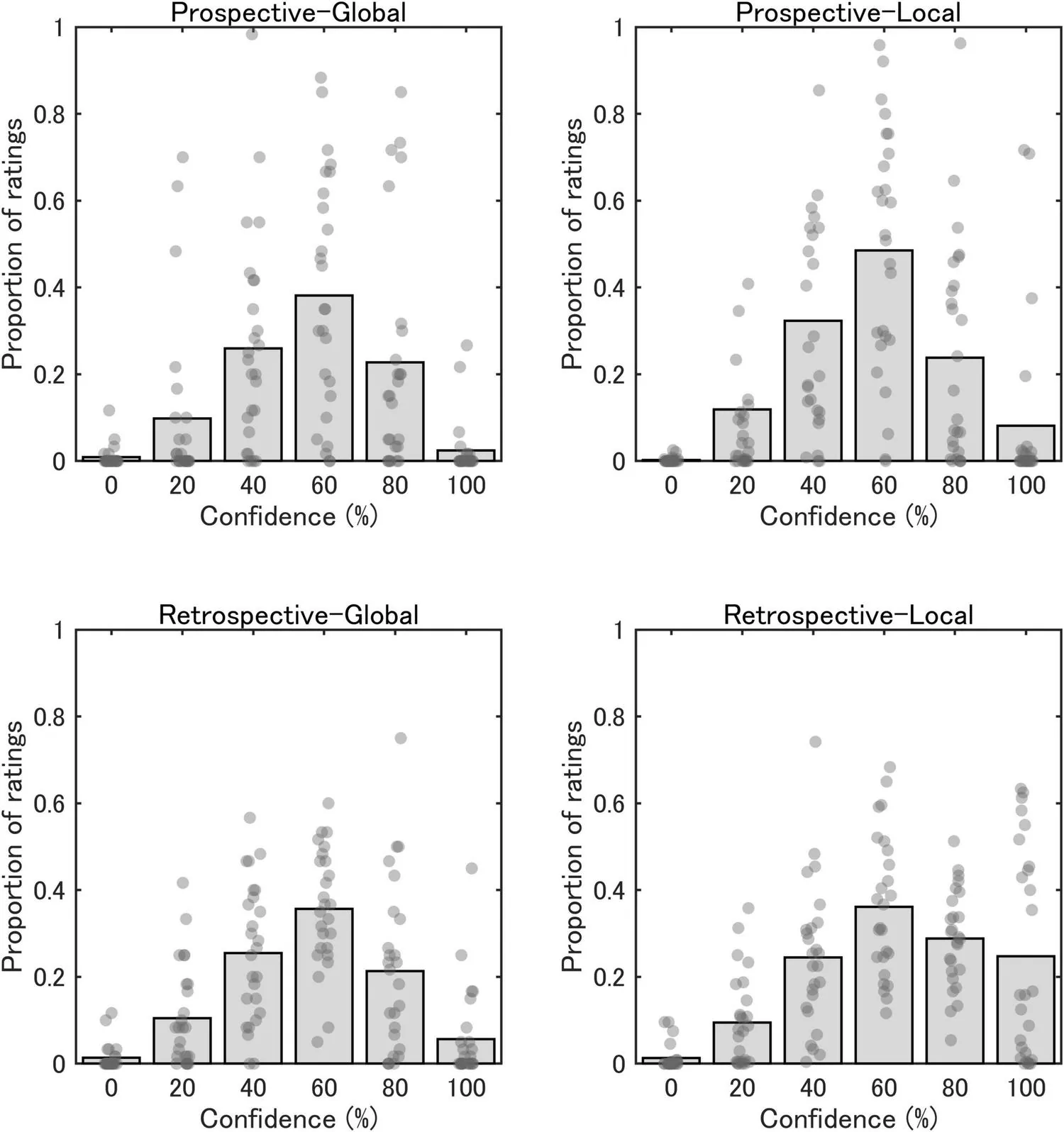
Distributions of confidence ratings across the four judgment conditions. Bars indicate the mean proportion of ratings at each confidence level across participants, and individual gray dots indicate the corresponding proportion for each participant.

Metacognitive bias was analyzed using a linear mixed-effects model with temporal focus (prospective vs. retrospective), temporal scope (global vs. local), and their interaction as fixed effects and a random intercept for participant. There was a significant main effect of temporal focus, with less negative bias in the retrospective than in the prospective condition (β = 0.046, *SE* = 0.015, *t*(100) = 3.112, *p* = 0.002, 95% CI [0.017, 0.075]). There was also a significant main effect of temporal scope, with less negative bias in the local than in the global condition (β = 0.048, *SE* = 0.015, *t*(100) = 3.273, *p* = 0.001, 95% CI [0.019, 0.077]). The interaction between temporal focus and temporal scope was not significant (β = 0.012, *SE* = 0.029, *t*(100) = 0.406, *p* = 0.686, 95% CI [−0.046, 0.070]).

One-sample *t*-tests against zero confirmed that metacognitive bias was significantly below zero in all conditions [prospective–global: *t*(25) = −6.057, *p* < 0.001, *d* = −1.188; prospective–local: *t*(25) = −4.911, *p* < 0.001, *d* = −0.963; retrospective–global: *t*(25) = −6.049, *p* < 0.001, *d* = −1.186; retrospective–local: *t*(25) = −3.937, *p* < 0.001, *d* = −0.772]. Thus, metacognitive bias was consistently negative across all four conditions, indicating underconfidence (Figure 4).

**FIGURE 4:**
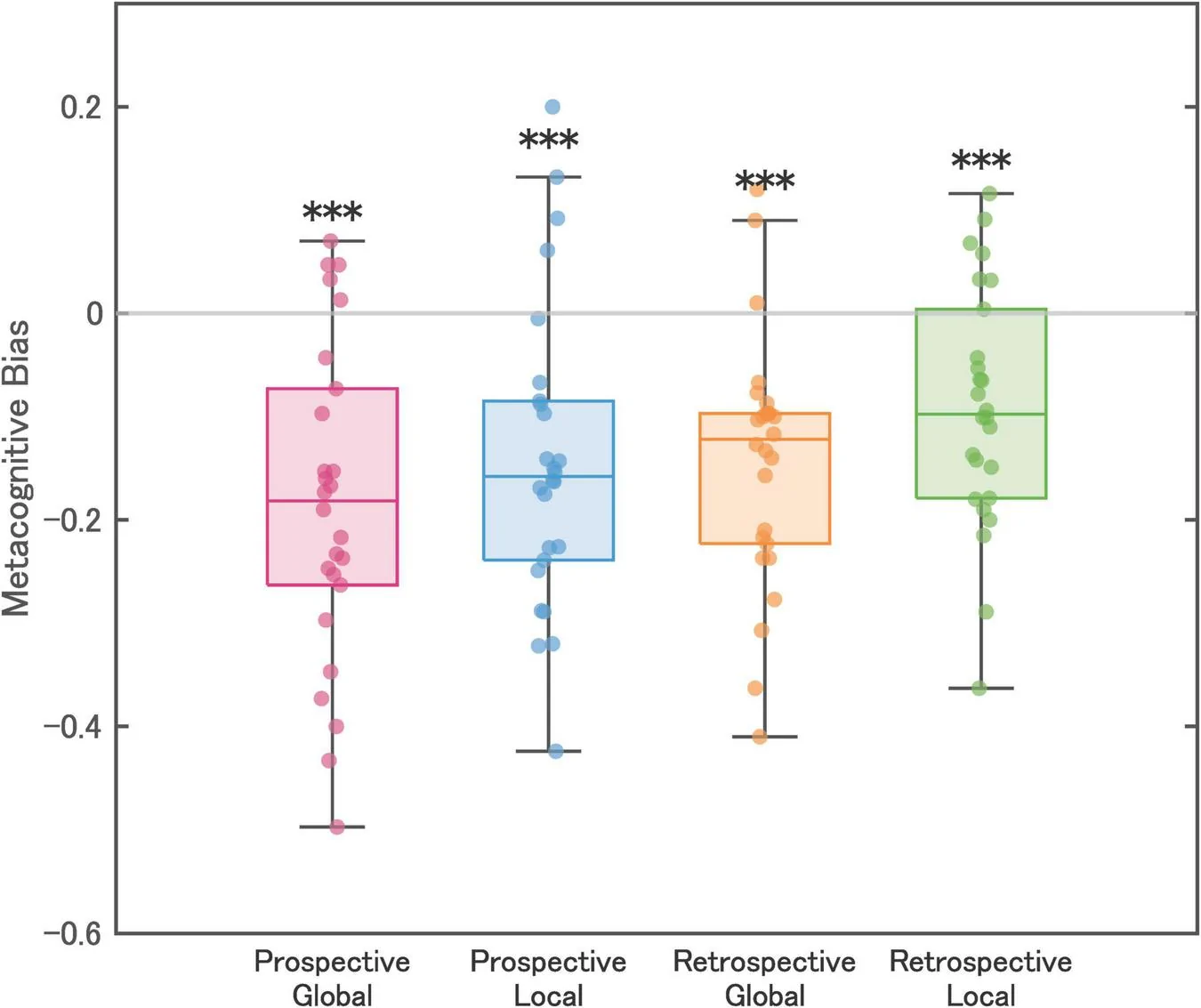
Metacognitive bias for prospective and retrospective metacognitive judgments in the global and local conditions. Negative values indicate underconfidence. Boxes indicate the interquartile range (IQR), center lines indicate medians, whiskers indicate values within 1.5 × IQR, and dots represent individual participants. Asterisks indicate significant differences from zero, based on one-sample *t*-tests (***p < 0.001).

## 4 Discussion

This study compared prospective and retrospective metacognitive judgments at global and local levels in a perceptual detection task, with a focus on how confidence–performance relationships differed as a function of temporal focus and scope under different informational conditions. By separating within-participant confidence variation from participant-condition mean differences, the present analyses also distinguished two levels at which confidence may be associated with task performance.

The results demonstrate that prospective confidence was positively associated with task performance at the participant-condition mean level: participants with higher mean confidence within a condition tended to show higher accuracy. However, within-participant confidence–performance associations were not statistically significant in either prospective condition. In contrast, within-participant associations were positive and significant in both retrospective conditions and were strongest under local evaluations. Consistent with previous research (Fleming et al., 2016; Siedlecka et al., 2016; Zacharopoulos et al., 2022), this pattern suggests that retrospective metacognition benefits from access to decision-related evidence that is not fully available at the prospective stage. Complementary AUROC2 and Goodman–Kruskal’s gamma analyses were consistent with the absence of statistically significant within-participant prospective associations, although these nonsignificant results do not establish that prospective sensitivity is absent.

Previous studies have examined prospective metacognition under different informational conditions. For example, Fleming et al. (2016) asked participants to predict their performance before every fifth upcoming trial, allowing them to draw on information accumulated from previous trials, whereas Siedlecka et al. (2016) compared prospective judgments made before and after presentation of the target stimulus. In contrast, in the present study, prospective confidence was assessed before presentation of the upcoming stimulus and before the corresponding Type-1 response, without providing trial-specific cues about the upcoming stimulus. Thus, the present findings extend previous work by showing that a positive association between prospective confidence and performance at the participant-condition mean level can coexist with nonsignificant within-participant associations. The positive association in the original pooled analysis should therefore not be interpreted as evidence that participants could reliably distinguish upcoming occasions on which they would perform better from those on which they would perform worse.

In perceptual decision making, retrospective confidence has been shown to incorporate decision-related internal signals, such as the strength of accumulated evidence and response fluency (Kiani et al., 2014; Pouget et al., 2016). As such signals are generated during or after the decision process, they are not available when individuals are required to predict their performance before a stimulus presentation. Consistent with this view, confidence judgments made after a response are typically more accurate than those made prospectively (Fleming et al., 2016; Siedlecka et al., 2016), suggesting that the decision process itself provides additional diagnostic information. Taken together, these findings are compatible with informational constraints on prospective metacognition, but do not directly establish that such constraints caused the observed differences.

The distinction between the two confidence components is also important for interpreting the positive participant-condition mean associations, which were observed in all four conditions. These associations indicate that differences in average confidence between participants corresponded to differences in task accuracy within each condition. They may reflect general expectations about performance, differences in task ability, or other participant characteristics associated with both confidence and accuracy. However, they do not establish that an individual’s confidence tracked fluctuations in their own performance across observations. Nor does a positive association between mean confidence and accuracy necessarily imply accurate calibration of confidence. No pairwise differences between the participant-condition mean slopes were significant after correction, although this does not establish that the slopes were equivalent across conditions.

The additional analyses examining the information underlying prospective confidence provide further insight into this interpretation. Within participants, prospective confidence was consistently associated with recent local confidence and confidence from the preceding global observation. Associations with previous task accuracy were more limited: recent local accuracy was positively associated with prospective-local confidence, whereas the remaining within-participant accuracy-history coefficients were not significant after Holm correction. This pattern is compatible with the possibility that prospective confidence incorporates information accumulated from prior metacognitive experiences. However, these observational associations could also reflect persistent response tendencies or other shared influences on successive confidence judgments.

Furthermore, none of the examined within-participant history predictors was significantly associated with subsequent task performance in either prospective condition after correction. Taken together, prospective confidence was associated with recent confidence history, whereas the examined historical information did not show significant associations with subsequent performance. This pattern is consistent with the possibility that prospective confidence reflects an ongoing summary of recent experience rather than a direct readout of diagnostic information about the upcoming judgment target. These analyses do not, however, identify the causal cues used to form confidence or demonstrate that the examined historical information has no predictive value. Attentional or motivational fluctuations remain possible influences that were not directly measured in the present study.

The GLMM results also provide insights into the distinction between global and local metacognitive judgments. The within-participant confidence–performance relationship was the strongest for retrospective judgments in the local condition, whereas the corresponding relationship was weaker for retrospective judgments in the global condition. This pattern suggests that the trial-specific internal signals available after each perceptual decision may be especially informative when confidence is evaluated at the local level. In contrast, such signals may be less directly applicable to global retrospective judgments, which require observers to evaluate performance over multiple trials, rather than in a single trial.

Unlike retrospective confidence, prospective confidence showed no significant difference in within-participant slopes between global and local conditions, and neither prospective slope differed significantly from zero. This pattern is compatible with the possibility that even local prospective judgments lacked sufficiently diagnostic information about the upcoming stimulus or performance episode to produce a local advantage. One possibility is that prospective confidence reflected more general expectations of task difficulty or anticipated performance. Although prospective global and local judgments may have drawn on overlapping sources of information, they concerned different targets: performance across the next five trials and performance on a single upcoming trial, respectively. Thus, the absence of a significant slope difference does not demonstrate that prospective global and local judgments were psychologically identical or relied on exactly the same information.

This interpretation is consistent with recent work suggesting that local and global metacognitive judgments are not simply different summaries of the same confidence signal. For example, Seow et al. (2021) demonstrate that global self-performance estimates vary independently of mean local confidence, indicating that they capture information beyond aggregated trial-level evaluations. Similarly, Rouault and Fleming (2020) proposed that global self-beliefs operate as higher-order priors that integrate information over extended timescales, rather than reflecting a simple summation of momentary confidence signals. Prospective confidence in the present study may therefore have relied on information less directly tied to the outcome of the upcoming judgment target than the signals supporting retrospective confidence.

This interpretation is also consistent with research on judgments of learning (JOLs), which has shown that prospective evaluations are influenced by belief-based cues–such as theories about one’s ability or task familiarity–rather than solely by experience-based information (Koriat, 1997; Dunlosky and Metcalfe, 2008). Although JOLs have typically been studied in the context of memory, they provide a useful parallel for understanding prospective confidence in perceptual decision making. Specifically, they suggest that prospective metacognitive judgments may draw on belief-based or contextual cues that are partially independent of recently accumulated trial-level evidence. Overall, the present findings are consistent with the possibility that weaker prospective metacognitive sensitivity reflects limitations in accessible decision-related information. However, they do not resolve whether informational constraints or other differences in metacognitive processing account for the observed pattern. By systematically comparing prospective and retrospective judgments across global and local levels, this study highlights the importance of distinguishing within-participant sensitivity from associations between participants’ mean confidence and performance.

An additional perspective concerns the brain mechanisms underlying prospective and retrospective metacognitive judgments. Previous neuroimaging studies have implicated a distributed network of prefrontal and medial frontal regions in metacognitive monitoring, including lateral and rostrolateral prefrontal cortex and posterior medial frontal regions (e.g., Fleming et al., 2010; Vaccaro and Fleming, 2018). More directly relevant to prospective metacognition, Miyamoto et al. (2021) showed that anterior lateral prefrontal cortex represented information used to predict future perceptual performance, and that disrupting this region impaired prospective metacognitive judgments without affecting perceptual performance itself. Recent work using noninvasive brain stimulation has further highlighted the potential for causal investigation of second-order judgments. Saccenti et al. (2024) reviewed this literature from the perspective of prospective and retrospective metacognition, and Saccenti et al. (2025) reported that transcranial direct current stimulation over the orbitofrontal cortex altered self-reported confidence and reduced metacognitive sensitivity in a perceptual decision-making task, while leaving first-order perceptual accuracy unaffected. Together, these findings indicate that metacognitive judgments can be influenced by modulation of specific brain regions. However, a clear neural dissociation between prospective and retrospective metacognition has not yet been established. The behavioral distinction between prospective and retrospective judgments provides a basis for future work examining whether modulation of candidate brain regions differentially affects prospective and retrospective metacognitive sensitivity or bias, and whether such effects vary between global and local judgments.

Metacognitive bias was negative in all conditions, indicating a general tendency toward underconfidence. This tendency was stronger for prospective and global judgments, which may reflect more conservative confidence when judgments are less directly supported by trial-specific diagnostic information. This interpretation is consistent with the concept of retrospective metacognitive judgments drawing on additional post-decision and trial-specific cues that are prospectively unavailable. These bias results complement the decomposition analysis: participants with higher mean confidence could show higher accuracy while nevertheless underestimating their performance on average. Thus, the positive participant-condition mean associations should not be interpreted as evidence of unbiased confidence. As prior work on prospective and retrospective metacognition focused mainly on accuracy-related aspects of performance monitoring, the present results extend the literature by also highlighting systematic differences in metacognitive bias.

The distinction between prospective and retrospective metacognition may also be relevant to metacognitive alterations associated with mental health. Previous research has linked psychiatric symptoms to differences in confidence and metacognitive sensitivity (Rouault et al., 2018; Hohendorf and Bauer, 2023). Although this literature has primarily focused on retrospective metacognition, future research could examine whether clinical populations show distinct patterns of prospective and retrospective metacognition across global and local conditions. Separating within-participant sensitivity, mean confidence, and metacognitive bias may provide a more comprehensive characterization of these patterns.

Despite its clear contributions, this study has several limitations. First, the present study did not directly manipulate the amount or type of information available for metacognitive judgments. Therefore, informational constraints should be regarded as an interpretation of the present pattern of results rather than a direct causal conclusion. The exploratory history analyses likewise establish associations rather than causal use of particular cues, and they do not rule out confidence-related changes in detection behavior. Second, the participant-level sample size was relatively small, which may limit the generalizability and precision of the participant-condition mean associations. The large number of observations per participant provided information about within-participant relationships, but did not increase the number of independent participants available for estimating between-participant associations. Moreover, nonsignificant prospective within-participant slopes should not be interpreted as evidence of zero sensitivity or equivalence between global and local conditions. Replication with a larger and more diverse sample is needed to assess the robustness of these findings. Third, global and local judgments differed not only in judgment level, but also in the structure of the performance outcome: global performance was based on aggregated accuracy across five trials, whereas local performance was based on single-trial correctness. This difference was inherent to the present design, but it complicates the direct interpretation of global–local differences. Future studies could further examine prospective and retrospective metacognition using designs that closely equate outcome structures across judgment levels.

In conclusion, the present study showed that prospective confidence was positively associated with task performance at the participant-condition mean level, whereas its within-participant associations were not statistically significant. Retrospective confidence showed positive within-participant associations in both global and local conditions, with a stronger association in the local condition. The additional analyses showed that prospective confidence was consistently related to recent confidence judgments within participants, but the examined historical information was not significantly associated with subsequent performance after correction. These findings are compatible with constraints on the information available to prospective judgments and emphasize the distinction between mean-level confidence–performance associations and within-participant metacognitive sensitivity. Future research should further clarify the cues that observers use to judge their future performance, how these cues differ from those supporting retrospective confidence.

## Data Availability

The datasets presented in this study can be found in online repositories. The name of the repository and the accession information are as follows: Open Science Framework (OSF), https://osf.io/up7bh/.

## Footnotes

1
https://chatgpt.com
