# Supplementary material for: Confidence–performance relationships in prospective and retrospective metacognition across local and global judgments
Source: Front Psychol. 2026 Oct 29;17:1900905. doi: 10.3389/fpsyg.2026.1900905 (PMC13755211; doi:10.3389/fpsyg.2026.1900905)
Supplement: Supplementary file 1 [file Supplementary_file_1.docx]

Supplementary Material

# 1 Supplementary Results

**1.1** **Task Performance and Target Gabor Contrast Across Blocks**

Table S1 presents the mean task accuracy and target Gabor contrast by block and block-order group. The corresponding mixed-design ANOVA results are reported in the main text.

### **Table S1.** Mean task accuracy and target Gabor contrast by block and block-order group.

| Block order | Block | Task accuracy | | Target Gabor contrast | |
| --- | --- | --- | --- | --- | --- |
|  |  | Mean | *SD* | Mean | *SD* |
| A First  (*N* = 13) | A | 0.742 | 0.071 | 42.385% | 7.107% |
|  | B | 0.745 | 0.068 | 34.538% | 4.090% |
| B First  (*N* = 13) | A | 0.702 | 0.058 | 35.308% | 4.465% |
|  | B | 0.715 | 0.069 | 35.215% | 5.322% |

***Note.*** A First = Block A was completed before Block B; B First = Block B was completed before Block A.

## 1.2　 AUROC2

AUROC2 was calculated separately for each participant and condition as a complementary measure of metacognitive sensitivity (Fleming & Lau, 2014). AUROC2 values range from 0 to 1, with 0.50 indicating chance-level discrimination between correct and incorrect responses based on confidence.

AUROC2 was analyzed using a linear mixed-effects model with temporal focus (prospective vs. retrospective), temporal scope (global vs. local), and their interaction as fixed effects and a random intercept for each participant. The LMM was specified using the following Wilkinson notation:

*AUROC2 ~ temporal focus * temporal scope + (1 | subject).*

One-sample *t*-tests were also conducted to determine whether AUROC2 values differed from 0.5 in each condition, with Holm correction applied across the four comparisons. AUROC2 did not significantly differ from 0.5 in either the prospective–global condition (*M* = .512, *t*(25) = 1.272, *p* = .297) or the prospective–local condition (*M* = .509, *t*(25) = 1.491, *p* = .297). In contrast, AUROC2 was significantly greater than 0.5 in both the retrospective–global condition (*M* = .588, *t*(25) = 5.252, *p* < .001) and the retrospective–local condition (*M* = .650, *t*(25) = 10.825, *p* < .001).

The LMM revealed a significant main effect of temporal focus, with higher AUROC2 in the retrospective than in the prospective condition (*β* = 0.105, *SE* = 0.013, *t*(100) = 8.333, *p* < .001, 95% CI [.0802, .1303]). There was also a significant main effect of temporal scope, with higher AUROC2 in the local than in the global condition (*β* = 0.026, *SE* = 0.013, *t*(100) = 2.062, *p* = .042, 95% CI [.0009, .0511]). The interaction between temporal focus and temporal scope was significant (*β* = 0.072, *SE* = 0.025, *t*(100) = 2.846, *p* = .005, 95% CI [.0218, .1220]). To further characterize this interaction, follow-up simple-effects tests with Holm correction showed that AUROC2 was higher in the retrospective than in the prospective condition for both global (*β* = 0.069, *SE* = 0.018, *t*(100) = 3.880, *p* < .001) and local judgments (*β* = 0.141, *SE* = 0.018, *t*(100) = 7.905, *p* < .001). The effect of temporal scope was not significant for prospective judgments (*β* = −0.010, *SE* = 0.018, *t*(100) = −0.553, *p* = .580), whereas AUROC2 was higher for local than global judgments in the retrospective condition (*β* = 0.062, *SE* = 0.018, *t*(100) = 3.471, *p* = .001).

**Figure S1.** AUROC2 for prospective and retrospective metacognitive judgments in the global and local conditions.

*Note.* Boxes show the IQR, center lines the medians, whiskers values within 1.5 × IQR, and dots individual participants. ****p* < .001 versus 0.5 (Holm-corrected).

## 1.3 Goodman–Kruskal’s Gamma

Goodman–Kruskal’s gamma was calculated separately for each participant and condition as a complementary measure of the association between confidence and task performance. Gamma values range from −1 to 1, with positive values indicating a positive association between confidence and task performance.

Gamma was analyzed using a 2 × 2 repeated-measures ANOVA, with temporal focus (prospective vs. retrospective) and temporal scope (global vs. local) as within-participant factors. One-sample *t*-tests were also conducted to determine whether gamma differed from zero in each condition, with Holm correction applied across the four comparisons.

The ANOVA revealed significant main effects of temporal focus (*F*(1, 25) = 28.161, *p* < .001) and temporal scope (*F*(1, 25) = 4.585, *p* = .042), as well as a significant interaction between temporal focus and temporal scope (*F*(1, 25) = 14.017, *p* < .001). Follow-up comparisons showed that gamma was lower for prospective than for retrospective judgments in both the global condition (*t*(25) = −2.463, *p* = .042) and the local condition (*t*(25) = −7.249, *p* < .001). Global and local judgments did not differ in the prospective condition (*t*(25) = 0.284, *p* = .78), whereas gamma was higher for local than for global judgments in the retrospective condition (*t*(25) = −3.783, *p* = .003).

One-sample *t*-tests showed that gamma did not significantly differ from zero in the prospective–global condition (*M* = .074, *t*(25) = 1.279, *p* = .374) or the prospective–local condition (*M* = .061, *t*(25) = 1.357, *p* = .374). In contrast, gamma was significantly greater than zero in the retrospective–global condition (*M* = .224, *t*(25) = 6.008, *p* < .001) and the retrospective–local condition (*M* = .419, *t*(25) = 11.426, *p* < .001).

**Figure S2.** Goodman–Kruskal’s gamma for prospective and retrospective metacognitive judgments in the global and local conditions.

*Note.* Boxes show the IQR, center lines the medians, whiskers values within 1.5 × IQR, and dots individual participants. ****p* < .001 versus 0 (Holm-corrected).

**1.4	Original Pooled GLMM of Confidence and Task Performance**

### For transparency, we report the original pooled GLMM used in the initial manuscript, in which confidence was not decomposed into within-participant and participant-condition mean components. The model used a binomial distribution with a logit link and included confidence, temporal focus, temporal scope, their interactions, block order, and participant-specific random intercepts and confidence slopes. Confidence was z-standardized using a single mean and standard deviation calculated across all original confidence-rating observations from all participants and conditions, before each global observation was expanded into five trial-level binary outcomes (correct vs. incorrect) for the GLMM. The model was specified as:

### *number of correct ~ confidence rating(z-score) * temporal focus * temporal scope + block order + (1 + confidence rating(z-score) | subject).*

### Here, “number of correct” denotes the binary correctness outcome for each trial-level row (0 = incorrect, 1 = correct), including the binary outcomes generated from each global observation. Confidence was entered without separating within-participant variation from participant-condition mean differences.

### Table S2 presents the complete fixed-effect estimates. At the prospective–global reference condition, confidence was positively associated with performance (*β* = 0.133, *SE* = 0.039, *z* = 3.362, *p* < .001). The confidence × temporal focus interaction was significant (*β* = 0.130, *SE* = 0.041, *z* = 3.162, *p* < .01), indicating a steeper confidence–performance slope for retrospective than prospective judgments at the global reference level. The three-way interaction among confidence, temporal focus, and temporal scope was also significant (*β* = 0.242, *SE* = 0.060, *z* = 4.026, *p* < .001), indicating that this slope difference varied across temporal scopes. Neither block order (*p* = .706) nor the confidence × temporal scope interaction at the prospective reference level (*p* = .525) was significant.

### **Table S2.** Full generalized linear mixed-effects logistic regression model predicting task performance.

| Predictor | *β* | *SE* | z | *OR* | 95% CI for *OR* | *p* |
| --- | --- | --- | --- | --- | --- | --- |
| Intercept | 1.106 | 0.063 | 17.597 | 3.023 | 2.673–3.420 | < .001 |
| Confidence | 0.133 | 0.039 | 3.362 | 1.142 | 1.057–1.234 | < .001 |
| Temporal focus  (prospective vs. retrospective) | -0.151 | 0.038 | -3.987 | 0.860 | 0.798–0.926 | < .001 |
| Temporal scope (global vs. local) | -0.150 | 0.040 | -3.767 | 0.861 | 0.796–0.931 | < .001 |
| Block order | 0.011 | 0.028 | 0.377 | 1.011 | 0.956–1.068 | .706 |
| Confidence × Temporal focus | 0.130 | 0.041 | 3.162 | 1.139 | 1.051–1.234 | < .010 |
| Confidence × Temporal scope | 0.028 | 0.044 | 0.636 | 1.028 | 0.943–1.122 | .525 |
| Temporal focus × Temporal scope | 0.236 | 0.057 | 4.147 | 1.266 | 1.132–1.415 | < .001 |
| Confidence × Temporal focus  × Temporal scope | 0.242 | 0.060 | 4.026 | 1.273 | 1.132–1.432 | < .001 |

*Note. β* = log-odds coefficient; *SE* = standard error; *OR* = odds ratio; CI = confidence interval. Prospective and global judgments were the reference levels. Confidence was standardized across all original observations. *p*-values and CIs are unadjusted.

## 1.5 Simple Slopes and Comparisons in the Original Pooled GLMM

### To interpret the interactions involving confidence, we estimated condition-specific confidence slopes and compared slopes across conditions. Figure S3 shows the model-predicted confidence–performance relationships from the original pooled GLMM. The simple slopes were positive and significant in all four conditions after Holm correction (Table S3).

### **Figure S3.** Model-predicted confidence–performance relationships from the original pooled GLMM.

### *Note.* Lines represent model predictions and shaded areas indicate 95% confidence intervals.

### **Table S3.** Simple slopes of confidence predicting task performance in the original pooled GLMM.

| Metacognitive condition | *β* | *SE* | *z* | *OR* | 95% CI for *OR* | Holm-  corrected *p* |
| --- | --- | --- | --- | --- | --- | --- |
| Prospective–global | 0.133 | 0.039 | 3.362 | 1.142 | 1.057–1.234 | < .001 |
| Prospective–local | 0.161 | 0.040 | 4.000 | 1.174 | 1.085–1.271 | < .001 |
| Retrospective–global | 0.262 | 0.035 | 7.406 | 1.300 | 1.213–1.394 | < .001 |
| Retrospective–local | 0.532 | 0.036 | 14.612 | 1.702 | 1.585–1.828 | < .001 |

### *Note. p*-values are Holm-adjusted across the four simple-slope tests; CIs are unadjusted.

### The slope comparisons reported in the original manuscript are presented in Table S4. Confidence–performance slopes were significantly shallower for prospective than retrospective judgments at both global and local levels. The retrospective–global slope was also significantly shallower than the retrospective–local slope. The prospective–global and prospective–local slopes did not significantly differ.

### **Table S4**. Selected comparisons of confidence slopes in the original pooled GLMM.

| Predictor | *β* difference | *SE* | z | *OR* | 95% CI for *OR* | Holm-  corrected *p* |
| --- | --- | --- | --- | --- | --- | --- |
| Prospective–global vs. Retrospective–global | -0.130 | 0.041 | -3.162 | 0.878 | 0.810–0.952 | .002 |
| Prospective–global vs. Prospective–local | -0.028 | 0.044 | -0.636 | 0.972 | 0.892–1.060 | .525 |
| Retrospective–global vs. Retrospective–local | -0.270 | 0.040 | -6.803 | 0.764 | 0.707–0.825 | < .001 |
| Prospective–local vs. Retrospective–local | -0.371 | 0.043 | -8.543 | 0.690 | 0.633–0.751 | < .001 |

### *Note.* Differences are calculated as the first condition minus the second. *p*-values are Holm-adjusted across the four comparisons; CIs are unadjusted.

### Although the original pooled model showed positive prospective confidence–performance associations, it did not distinguish within-participant variation from differences in participant-condition mean confidence. Therefore, these prospective slopes should not be interpreted as evidence of within-participant sensitivity to upcoming performance. In the primary decomposition GLMM, prospective participant-condition mean associations were positive and significant, whereas prospective within-participant associations were not statistically significant. The original pooled results are retained to document the earlier analysis and clarify the change in interpretation.

### 1.6 Exploratory Analyses of Judgment History

**1.6.1 History Predictors and Model Specifications**

These exploratory analyses examined whether current confidence was associated with previous confidence and task accuracy, and whether the same historical information was associated with performance on the current judgment target. Separate linear mixed-effects models (LMMs) of confidence and binomial generalized linear mixed-effects models (GLMMs) of performance were fitted for each condition. The prospective analyses supplement Section 3.3 of the main text; corresponding retrospective analyses were included to characterize associations with pre-target history.

Four history predictors were constructed: mean confidence and mean accuracy across the four most recent eligible local observations, and confidence and accuracy from the most recent eligible global observation. Accuracy was expressed as the proportion of correct responses for each source observation. The source conditions are summarized in Table S5.

**Table S5.** Sources of history predictors in each judgment condition.

| Current judgment condition | Recent local confidence and accuracy | Previous global confidence and accuracy |
| --- | --- | --- |
| Prospective–global | Four most recent eligible retrospective–local observations | Most recent completed prospective–global observation |
| Prospective–local | Four most recent eligible prospective–local observations | Most recent completed retrospective–global observation |
| Retrospective–global | Four most recent eligible prospective–local observations | Most recent completed retrospective–global observation |
| Retrospective–local | Four most recent eligible retrospective–local observations | Most recent completed prospective–global observation |

*Note.* Eligible observations were from the same participant and block and were completed before the current judgment target began. The four local observations were the four most recent eligible observations and were not necessarily consecutive physical trials.

The same temporal cutoff was applied to all four conditions. For retrospective–global judgments, local observations within the current five-trial target were excluded. For retrospective–local judgments, a prospective–global observation was excluded if its five-trial target had not yet been completed, even if its confidence rating had already been obtained. Consequently, the retrospective models examined associations with pre-target history rather than all information available at the time of the retrospective rating. This restriction also prevented historical accuracy predictors from including responses belonging to the current performance outcome.

Each history predictor was decomposed into within-participant and participant-condition mean components. The within-participant component was calculated by subtracting each participant’s condition-specific mean of the history predictor from its value at each observation. The participant-condition mean component was calculated by subtracting the mean of these participant-specific means from each participant’s mean. These means were calculated using the common complete-case sample for the confidence and performance models within each condition.

Both components of confidence-history predictors were divided by the pooled standard deviation of the original confidence ratings. Both components of accuracy-history predictors were divided by the pooled standard deviation of accuracy proportions in the original dataset. These scaling constants were calculated across participants and conditions before history-related exclusions. The dependent variable in the LMMs, current confidence, was z-standardized using the overall mean and standard deviation of the original confidence ratings. The following model structures were fitted separately for each condition:

**Confidence LMM:**

*current confidence_z ~ recent local confidence_W + recent local confidence_B + recent local accuracy_W + recent local accuracy_B + previous global confidence_W + previous global confidence_B + previous global accuracy_W + previous global accuracy_B + block order + (1 | subject)*

**Performance GLMM:**

*number of correct ~ recent local confidence_W + recent local confidence_B + recent local accuracy_W + recent local accuracy_B + previous global confidence_W + previous global confidence_B + previous global accuracy_W + previous global accuracy_B + block order + (1 | subject)*

Here, W and B denote the scaled within-participant and participant-condition mean components, respectively. All models included block order as a fixed effect and a participant random intercept. LMMs were fitted using restricted maximum likelihood. GLMMs used a binomial distribution and logit link, with a denominator of five for global observations and one for local observations, and were fitted using the Laplace approximation. Current confidence was not included as a predictor in the performance GLMMs.

The analyses included 26 participants in each condition. After complete-case exclusions, each global condition included 1,534 observations (59 per participant), and each local condition included 6,136 observations (236 per participant). The LMMs and GLMMs used the same observations within each condition.

Holm correction was applied separately by model type (LMM or GLMM), temporal-focus group (prospective or retrospective), and component (within-participant or participant-condition mean). Each correction family comprised eight tests: four history predictors across the two temporal-scope conditions. Intercept and block-order tests were not included in these families. Unless otherwise stated, *p*-values for history predictors are Holm-adjusted. Confidence intervals are unadjusted.

**1.6.2 Prospective Confidence and Performance**

In the prospective–global LMM, current confidence was positively associated with within-participant variations in recent retrospective–local confidence (*β* = 0.324, *SE* = 0.025, *t*(1524) = 13.172, *p* < .001) and previous prospective–global confidence (*β* = 0.143, *SE* = 0.024, *t*(1524) = 5.939, *p* < .001). Neither within-participant accuracy-history coefficient was significant after correction (both *ps* = .729).

In the prospective–local LMM, current confidence was positively associated with within-participant variations in recent prospective–local confidence (*β* = 0.526, *SE* = 0.017, *t*(6126) = 30.427, *p* < .001), previous retrospective–global confidence (*β* = 0.099, *SE* = 0.010, *t*(6126) = 9.756, *p* < .001), and recent prospective–local accuracy (*β* = 0.070, *SE* = 0.015, *t*(6126) = 4.523, *p* < .001). The within-participant coefficient for previous retrospective–global accuracy was not significant after correction (*β* = −0.040, *SE* = 0.017, *t*(6126) = −2.345, *p* = .057).

At the participant-condition mean level, previous prospective–global confidence was positively associated with current prospective–global confidence (*β* = 1.001, *SE* = 0.034, *p* < .001), and recent prospective–local confidence was positively associated with current prospective–local confidence (*β* = 0.999, *SE* = 0.016, *p* < .001). The remaining mean-component coefficients were not significant after correction. Because these two predictors summarize lagged values of the same confidence series as the dependent variable, their mean-level associations should not be interpreted as independent evidence of a temporal influence.

In the performance GLMMs, none of the within-participant history predictors was significant in either prospective condition (all *ps* ≥ .551). None of the participant-condition mean history predictors was significant after correction either (all *ps* ≥ .076). Thus, the analyses identified associations between prospective confidence and judgment history, but did not identify significant associations between the examined history predictors and subsequent performance after correction.

**1.6.3 Supplementary Analyses of Retrospective Judgments**

The same analyses were conducted for retrospective judgments using the condition-specific history sources shown in Table S5. As specified in Section 1.6.1, all history predictors were restricted to pre-target observations.

In the retrospective–global LMM, current confidence was positively associated with within-participant variations in recent prospective–local confidence (*β* = 0.228, *SE* = 0.045, *t*(1524) = 5.058, *p* < .001) and previous retrospective–global confidence (*β* = 0.154, *SE* = 0.027, *t*(1524) = 5.627, *p* < .001). Neither within-participant accuracy-history coefficient was significant after Holm correction (both *ps* = 1.000).

In the retrospective–local LMM, current confidence was positively associated with within-participant variations in recent retrospective–local confidence (*β* = 0.234, *SE* = 0.022, *t*(6126) = 10.484, *p* < .001). The coefficient for previous prospective–global confidence was not significant after correction (*β* = 0.040, *SE* = 0.022, *t*(6126) = 1.854, *p* = .319), and neither within-participant accuracy-history coefficient was significant (both *ps* = 1.000).

At the participant-condition mean level, previous retrospective–global confidence was positively associated with current retrospective–global confidence (*β* = 0.995, *SE* = 0.056, *p* < .001), and recent retrospective–local confidence was positively associated with current retrospective–local confidence (*β* = 0.990, *SE* = 0.039, *p* < .001). The remaining mean-component coefficients were not significant after correction. As in the prospective models, these significant mean-level confidence associations involved lagged summaries of the same confidence series as the dependent variable and should not be interpreted as independent evidence of a temporal influence. Full LMM results are presented in Table S6.

In the performance GLMMs, none of the within-participant history predictors was significant in either retrospective condition after correction (all *ps* = 1.000). At the participant-condition mean level, recent retrospective–local accuracy was positively associated with current retrospective–local performance (*β* = 2.213, *SE* = 0.712, *t*(6126) = 3.107, *p* = .015, *OR* = 9.144, 95% CI [2.263, 36.952]). The remaining mean-component coefficients were not significant after correction (all *ps* ≥ .144). Full GLMM results are presented in Table S7.

Thus, retrospective confidence was associated with aspects of pre-target confidence history, whereas the performance models identified a mean-level accuracy-history association in the retrospective–local condition but no significant within-participant history associations. These findings concern pre-target history and are distinct from the relationship between current retrospective confidence and performance examined in the primary decomposition GLMM. Differences in statistical significance across separately fitted condition-specific models do not establish differences between conditions.

**Table S6.** Fixed-effect estimates from the history LMMs predicting current confidence.

|  | Predictor | | *β* | *SE* | *t* | 95% CI for *β* | Holm-  corrected *p* |
| --- | --- | --- | --- | --- | --- | --- | --- |
|  | A. Prospective–global (*n* = 1,534 observations; *df* = 1,524) | | | | | | |
|  | Intercept | | −0.191 | 0.020 | −9.705 | −0.230 - −0.152 | < .001 |
|  | Block order | | −0.003 | 0.030 | −0.116 | −0.061 - 0.055 | .908 |
|  | Within-participant component | Recent local confidence | 0.324 | 0.025 | 13.172 | 0.276 - 0.372 | < .001 |
|  |  | Recent local accuracy | 0.031 | 0.057 | 0.537 | −0.081 - 0.142 | .729 |
|  |  | Previous global confidence | 0.143 | 0.024 | 5.939 | 0.096 - 0.190 | < .001 |
|  |  | Previous global accuracy | −0.057 | 0.062 | −0.907 | −0.179 - 0.066 | .729 |
|  | Participant-condition mean component | Recent local confidence | −0.006 | 0.043 | −0.137 | −0.089 - 0.078 | 1.000 |
|  |  | Recent local accuracy | −0.022 | 0.328 | −0.067 | −0.665 - 0.621 | 1.000 |
|  |  | Previous global confidence | 1.001 | 0.034 | 29.383 | 0.934 - 1.068 | < .001 |
|  |  | Previous global accuracy | 0.028 | 0.362 | 0.076 | −0.682 - 0.737 | 1.000 |
| B. Prospective–local (*n* = 6,136 observations; *df* = 6,126) | | | | | | | |
| Intercept | | | −0.135 | 0.010 | −13.773 | −0.154 - −0.116 | < .001 |
| Block order | | | 0.002 | 0.014 | 0.179 | −0.025 - 0.030 | 0.858 |
| Within-participant component | | Recent local confidence | 0.526 | 0.017 | 30.427 | 0.492 - 0.560 | < .001 |
|  |  | Recent local accuracy | 0.07 | 0.015 | 4.523 | 0.040 - 0.100 | < .001 |
|  |  | Previous global confidence | 0.099 | 0.010 | 9.756 | 0.079 - 0.118 | < .001 |
|  |  | Previous global accuracy | −0.040 | 0.017 | −2.345 | −0.074 - −0.007 | 0.057 |
| Participant-condition mean component | | Recent local confidence | 0.999 | 0.016 | 60.788 | 0.966 - 1.031 | < .001 |
|  |  | Recent local accuracy | 0.061 | 0.244 | 0.25 | −0.418 - 0.540 | 1.000 |
|  |  | Previous global confidence | 0.002 | 0.020 | 0.116 | −0.036 - 0.041 | 1.000 |
|  |  | Previous global accuracy | −0.059 | 0.252 | −0.233 | −0.553 - 0.435 | 1.000 |
| C. Retrospective–global (*n* = 1,534 observations; *df* = 1,524) | | | | | | | |
| Intercept | | | −0.165 | 0.028 | −5.982 | −0.219 - −0.111 | < .001 |
| Block order | | | −0.004 | 0.039 | −0.093 | −0.081 - 0.074 | 0.926 |
| Within-participant component | | Recent local confidence | 0.228 | 0.045 | 5.058 | 0.140 - 0.317 | < .001 |
|  |  | Recent local accuracy | −0.006 | 0.080 | −0.073 | −0.163 - 0.152 | 1.000 |
|  |  | Previous global confidence | 0.154 | 0.027 | 5.627 | 0.100 - 0.208 | < .001 |
|  |  | Previous global accuracy | −0.030 | 0.088 | −0.339 | −0.202 - 0.142 | 1.000 |
| Participant-condition mean component | | Recent local confidence | 0.008 | 0.046 | 0.183 | −0.082 - 0.098 | 1.000 |
|  |  | Recent local accuracy | −0.049 | 0.666 | −0.073 | −1.356 - 1.258 | 1.000 |
|  |  | Previous global confidence | 0.995 | 0.056 | 17.821 | 0.885 - 1.104 | < .001 |
|  |  | Previous global accuracy | 0.042 | 0.689 | 0.06 | −1.310 - 1.393 | 1.000 |
| D. Retrospective–local (*n* = 6,136 observations; *df* = 6,126) | | | | | | | |
| Intercept | | | 0.224 | 0.018 | 12.476 | 0.189 - 0.259 | < .001 |
| Block order | | | −0.004 | 0.027 | −0.134 | −0.056 - 0.049 | 0.894 |
| Within-participant component | | Recent local confidence | 0.234 | 0.022 | 10.484 | 0.190 - 0.278 | < .001 |
|  |  | Recent local accuracy | 0.001 | 0.028 | 0.042 | −0.053 - 0.055 | 1.000 |
|  |  | Previous global confidence | 0.04 | 0.022 | 1.854 | −0.002 - 0.083 | 0.319 |
|  |  | Previous global accuracy | −0.012 | 0.030 | −0.396 | −0.070 - 0.046 | 1.000 |
| Participant-condition mean component | | Recent local confidence | 0.99 | 0.039 | 25.3 | 0.913 - 1.067 | < .001 |
|  |  | Recent local accuracy | −0.006 | 0.291 | −0.020 | −0.577 - 0.565 | 1.000 |
|  |  | Previous global confidence | 0.006 | 0.031 | 0.188 | −0.055 - 0.067 | 1.000 |
|  |  | Previous global accuracy | 0.023 | 0.319 | 0.073 | −0.602 - 0.648 | 1.000 |

*Note.* History predictors are defined in Table S5. *p*-values for history predictors are Holm-adjusted as described in Section 1.6.1; intercept and block-order *p*-values and all CIs are unadjusted. *SE* = standard error; CI = confidence interval.

**Table S7.** Fixed-effect estimates from the history GLMMs predicting task performance.

|  | Predictor | | *β* | *SE* | | *t* | OR | | 95% CI for OR | Holm-  corrected *p* |
| --- | --- | --- | --- | --- | --- | --- | --- | --- | --- | --- |
|  | A. Prospective–global (*n* = 1,534 observations; *df* = 1,524) | | | | | | | | | |
|  | Intercept | | 1.105 | 0.041 | | 27.147 | 3.021 | | 2.789 - 3.272 | < .001 |
|  | Block order | | −0.006 | 0.061 | | −0.104 | 0.994 | | 0.881 - 1.120 | 0.917 |
|  | Within-participant component | Recent local confidence | −0.057 | 0.050 | | −1.146 | 0.944 | | 0.856 - 1.042 | 1.000 |
|  |  | Recent local accuracy | 0.209 | 0.115 | | 1.821 | 1.233 | | 0.984 - 1.544 | .551 |
|  |  | Previous global confidence | 0.065 | 0.049 | | 1.332 | 1.067 | | 0.970 - 1.174 | 1.000 |
|  |  | Previous global accuracy | −0.079 | 0.127 | | −0.623 | 0.924 | | 0.721 - 1.184 | 1.000 |
|  | Participant-condition mean component | Recent local confidence | −0.003 | 0.086 | | −0.036 | 0.997 | | 0.841 - 1.181 | 1.000 |
|  |  | Recent local accuracy | 0.207 | 0.649 | | 0.319 | 1.23 | | 0.345 - 4.390 | 1.000 |
|  |  | Previous global confidence | 0.003 | 0.067 | | 0.038 | 1.003 | | 0.879 - 1.144 | 1.000 |
|  |  | Previous global accuracy | 1.849 | 0.712 | | 2.596 | 6.351 | | 1.571 - 25.672 | 0.076 |
| B. Prospective–local (*n* = 6,136 observations; *df* = 6,126) | | | | | | | | | | |
| Intercept | | | 0.928 | | 0.041 | 22.771 | | 2.53 | 2.335 - 2.740 | < .001 |
| Block order | | | 0.018 | | 0.058 | 0.314 | | 1.018 | 0.909 - 1.141 | 0.754 |
| Within-participant component | | Recent local confidence | 0.111 | | 0.071 | 1.555 | | 1.118 | 0.971 - 1.286 | 0.840 |
|  |  | Recent local accuracy | −0.010 | | 0.064 | −0.159 | | 0.99 | 0.874 - 1.121 | 1.000 |
|  |  | Previous global confidence | 0.009 | | 0.042 | 0.212 | | 1.009 | 0.929 - 1.095 | 1.000 |
|  |  | Previous global accuracy | 0.096 | | 0.071 | 1.354 | | 1.1 | 0.958 - 1.263 | 1.000 |
| Participant-condition mean component | | Recent local confidence | −0.015 | | 0.066 | −0.222 | | 0.985 | 0.866 - 1.122 | 1.000 |
|  |  | Recent local accuracy | 2.08 | | 0.999 | 2.082 | | 8.002 | 1.129 - 56.705 | 0.262 |
|  |  | Previous global confidence | 0.018 | | 0.081 | 0.221 | | 1.018 | 0.869 - 1.193 | 1.000 |
|  |  | Previous global accuracy | −0.007 | | 1.030 | −0.007 | | 0.993 | 0.132 - 7.478 | 1.000 |
|  | C. Retrospective–global (*n* = 1,534 observations; *df* = 1,524) | | | | | | | | | |
|  | Intercept | | 0.900 | | 0.036 | 24.759 | | 2.458 | 2.289 - 2.640 | < .001 |
|  | Block order | | 0.020 | | 0.052 | 0.391 | | 1.021 | 0.921 - 1.130 | 0.696 |
|  | Within-participant component | Recent local confidence | 0.085 | | 0.059 | 1.435 | | 1.088 | 0.969 - 1.222 | 1.000 |
|  |  | Recent local accuracy | −0.056 | | 0.104 | −0.535 | | 0.946 | 0.771 - 1.160 | 1.000 |
|  |  | Previous global confidence | 0.006 | | 0.036 | 0.158 | | 1.006 | 0.937 - 1.079 | 1.000 |
|  |  | Previous global accuracy | 0.126 | | 0.114 | 1.109 | | 1.134 | 0.908 - 1.417 | 1.000 |
|  | Participant-condition mean component | Recent local confidence | −0.024 | | 0.058 | −0.412 | | 0.976 | 0.871 - 1.095 | 1.000 |
|  |  | Recent local accuracy | −0.053 | | 0.867 | −0.061 | | 0.949 | 0.173 - 5.193 | 1.000 |
|  |  | Previous global confidence | 0.032 | | 0.072 | 0.446 | | 1.033 | 0.896 - 1.190 | 1.000 |
|  |  | Previous global accuracy | 2.081 | | 0.898 | 2.318 | | 8.011 | 1.377 - 46.602 | 0.144 |
|  | D. Retrospective–local (*n* = 6,136 observations; *df* = 6,126) | | | | | | | | | |
|  | Intercept | | 1.118 | | 0.046 | 24.347 | | 3.06 | 2.796 - 3.348 | < .001 |
|  | Block order | | −0.018 | | 0.069 | −0.268 | | 0.982 | 0.858 - 1.123 | 0.789 |
|  | Within-participant component | Recent local confidence | −0.045 | | 0.056 | −0.806 | | 0.956 | 0.856 - 1.067 | 1.000 |
|  |  | Recent local accuracy | 0.101 | | 0.068 | 1.49 | | 1.106 | 0.969 - 1.264 | 1.000 |
|  |  | Previous global confidence | 0.081 | | 0.054 | 1.493 | | 1.084 | 0.975 - 1.205 | 1.000 |
|  |  | Previous global accuracy | 0.052 | | 0.073 | 0.706 | | 1.053 | 0.912 - 1.216 | 1.000 |
|  | Participant-condition mean component | Recent local confidence | 0.015 | | 0.096 | 0.155 | | 1.015 | 0.840 - 1.226 | 1.000 |
|  |  | Recent local accuracy | 2.213 | | 0.712 | 3.107 | | 9.144 | 2.263 - 36.952 | 0.015 |
|  |  | Previous global confidence | −0.007 | | 0.075 | −0.089 | | 0.993 | 0.857 - 1.151 | 1.000 |
|  |  | Previous global accuracy | −0.126 | | 0.775 | −0.162 | | 0.882 | 0.193 - 4.030 | 1.000 |

*Note*. History predictors are defined in Table S5. *p*-values for history predictors are Holm-adjusted as described in Section 1.6.1; intercept and block-order *p*-values and all CIs are unadjusted. *SE* = standard error; *OR* = odds ratio; CI = confidence interval.
